# Supplementary figures and images for: The CDT of Helicobacter hepaticus induces pro-survival autophagy and nucleoplasmic reticulum formation concentrating the RNA binding proteins UNR/CSDE1 and P62/SQSTM1
Source: PLoS Pathog. 2021 Mar 4;17(3):e1009320. doi: 10.1371/journal.ppat.1009320 (PMC7963068; doi:10.1371/journal.ppat.1009320)

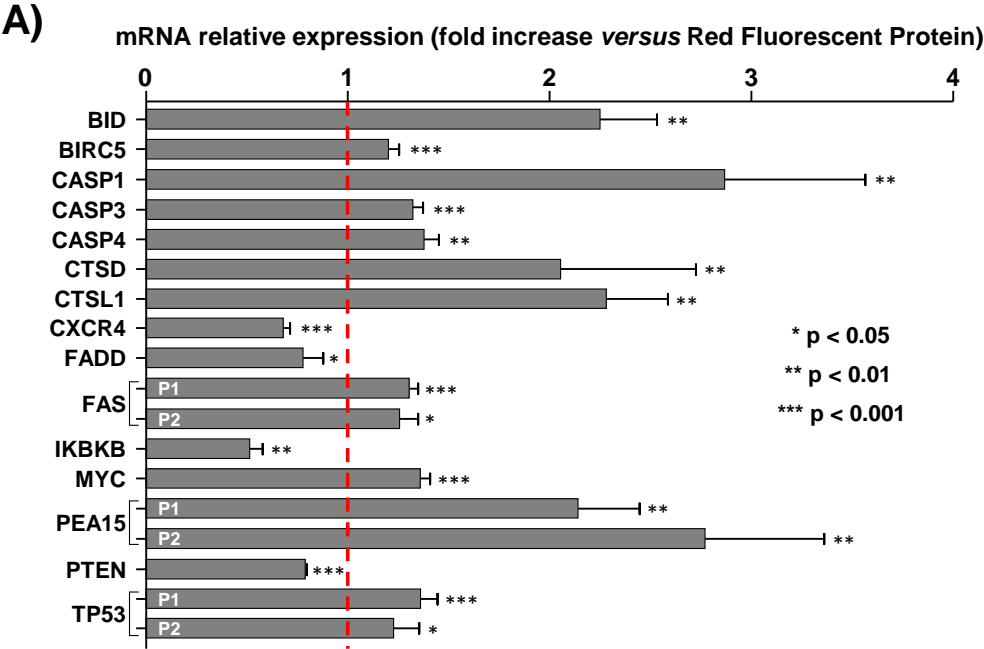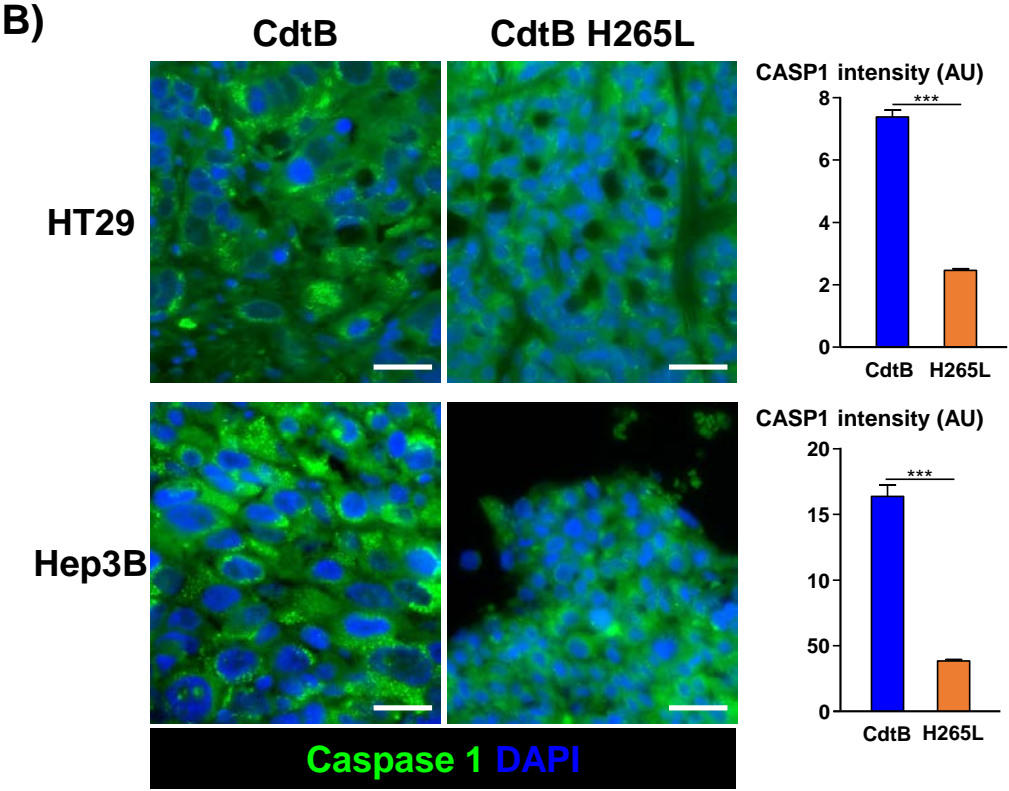

Supplement: S1 Fig — A) Microarray-based identification of differentially expressed autophagy associated apoptosis-related genes in response to Helicobacter hepaticus CdtB in HT29 intestinal epithelial cells. The gene expression, transduction protocol, relative gene expression, results presentation and gene selection are described in the legend of Fig 1. Asterisks denote significant results. P1 and P2 represent the 2 probe names (S3 Table) used for mRNA quantification. The data presented for BIRC5, CASP3, CXCR4, FAS (probe 1), MYC, PTEN and TP53 (probe 1) are the results of 40 replicates as 10 probes for each mRNA were included on the Microarray Kit. Details are presented in S3 Table (name and sequence of the probes, the corresponding gene name, the genbank accession number, the locus and the transcript variant). B) Images of 3 μm-tissue sections of HT-29- and Hep3B- derived mice engrafted tumors stained with fluorescent primary antibody to detect Caspase 1 (green) and DAPI to counterstain the nuclei (blue). Caspase 1 was quantified on a minimum of 200 cells using the "Integrated Density" measure function of ImageJ. Scale bar, 30 μm. ***p< 0.0001 versus H265L. Abbreviations: AU, arbitrary unit; BID, BH3 Interacting Domain death agonist; BIRC5, Baculoviral IAP Repeat-Containing 5; CASP, Caspase apoptosis-related cysteine peptidase; CTSD, Cathepsin D; CTSL1, Cathepsin L1; CXCR4, Chemokine (C-X-C motif) Receptor 4; DAPI, 4′, 6′-diamidino-2-phenylindol; FADD, Fas (TNFRSF6)-associated via death domain; FAS, Fas (TNF receptor superfamily, member 6); IKBKB, Inhibitor of Kappa light polypeptide gene enhancer in B-cells, Kinase Beta; MYC, v-myc myelocytomatosis viral oncogene homolog; PEA15, Phosphoprotein Enriched in Astrocytes 15; PTEN, Phosphatase and Tensin homolog; TP53, Tumor Protein p53. (PDF) [file ppat.1009320.s004.pdf]

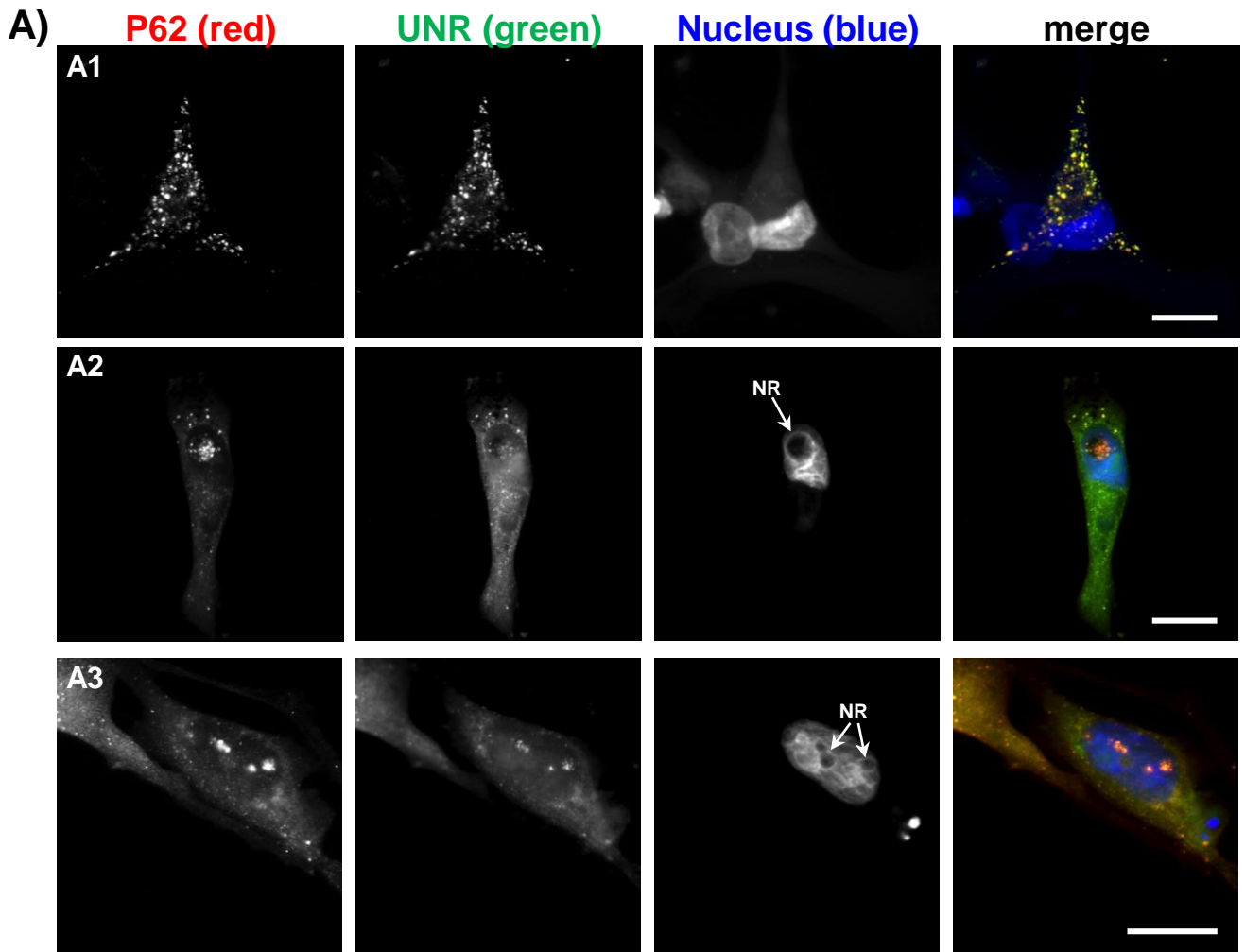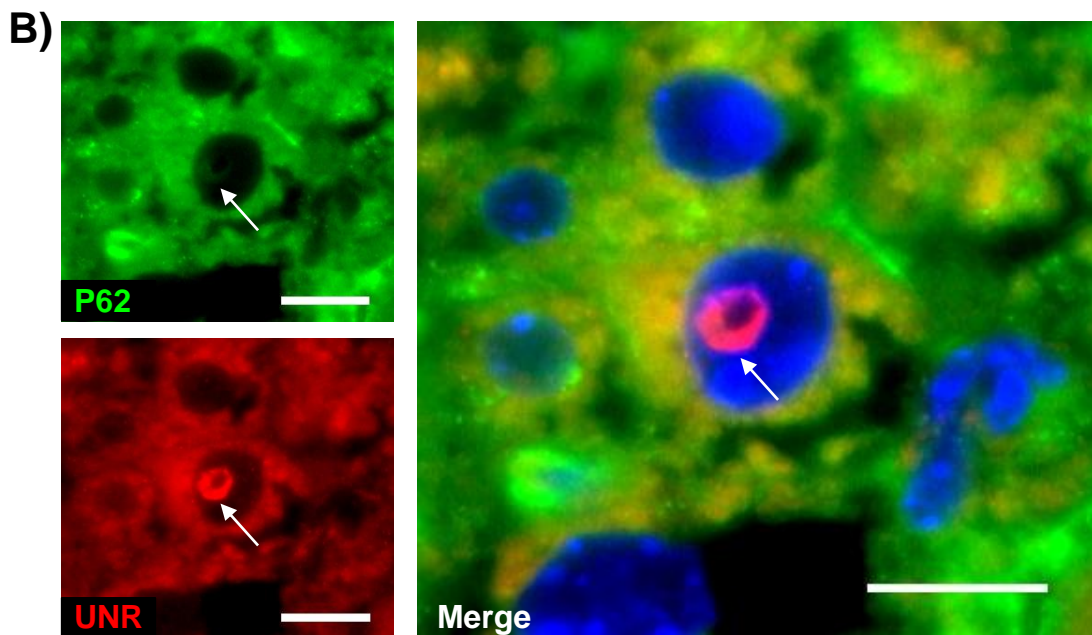

Supplement: S3 Fig — A) Images of SK-Hep-1 cells following a 72 h coculture with colibactin-producing Escherichia coli. SK-Hep-1 cells were non-infected or infected for 3 days with colibactin-secreting extra-intestinal pathogenic E. coli and its corresponding isogenic mutant. Then, cells were processed for fluorescent staining with primary antibodies generated against P62/SQSTM1 (red) and UNR/CSDE1 (green) associated with fluorescent labeled-secondary antibodies and DAPI to counterstain the nuclei (blue). SK-Hep-1 cells non infected and infected with E. coli that don’t secretes colibactin did not show increase P62/SQSTM1 bodies. Thus, only SK-Hep-1 cells infected with colibactin-secreting E. coli are shown. (A1) SK-Hep-1 cells with distended nucleus without NR formation. (A2) and (A3) SK-Hep-1 cells with distended nucleus with NR formation. Arrows indicate nucleoplasmic reticulum (NR). Scale bar, 30 μm. As previously demonstrated, NR formation is primarily observed in response to bacterial genotoxin, CDT and colibactin [6]. Thus, images of non-infected cells and cells infected with colibactine-defective mutant strain are not presented. (B) In vivo detection of nuclear remodeling following a 14 months infection with H. hepaticus. Non-transgenic mice were infected with H. hepaticus wild type strain 3B1for 14 months [23]. Mice livers were processed and stained as previously reported [6,23]. Image of tissue sections of infected liver stained with primary and fluorescent secondary antibodies: UNR (red), P62/SQSTM1 (green) and DAPI to counterstain the nuclei (blue). The zone without UNR/P62/DAPI staining in the core of NR probably corresponds to a mitochondrion, as previously reported [6]. P62/SQSTM1-labeling generated a significant background noise in the mice liver tumor infiltrates, preventing efficient quantification. As previously demonstrated, NR formation is primarily observed in response to CDT intoxication, via its active CdtB subunit [6]. Thus, images of non-infected mice are not pr [file ppat.1009320.s006.pdf]

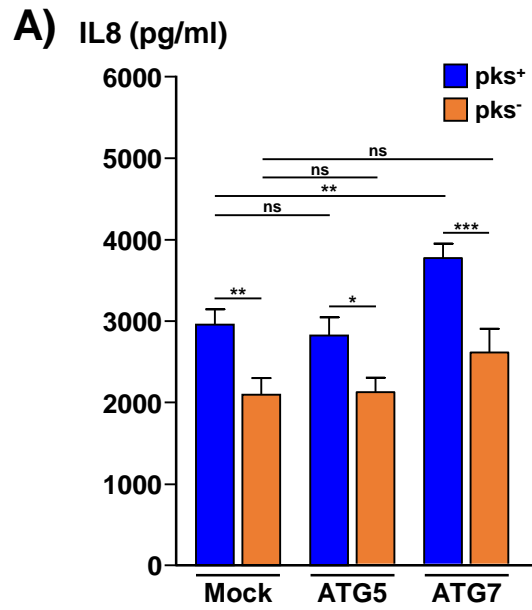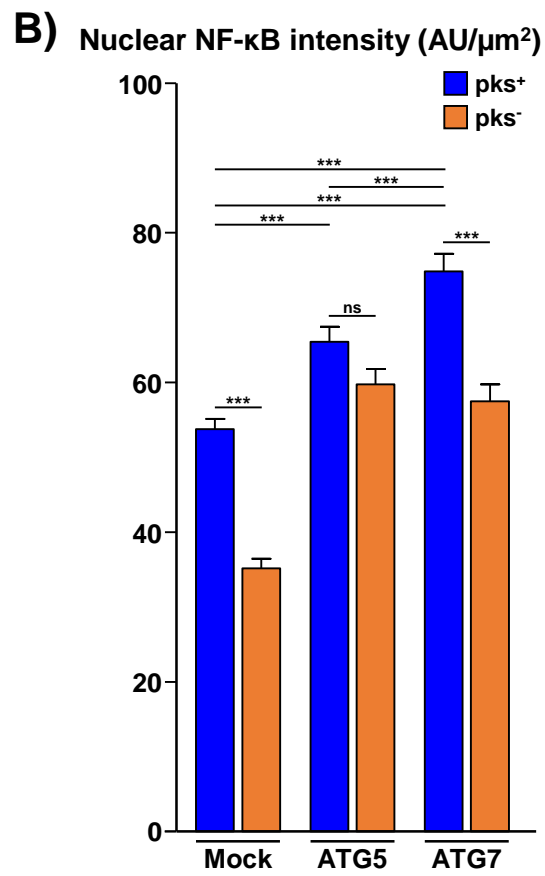

Supplement: S4 Fig — Mock-KO, ATG5-KO and ATG7-KO Hep3B cells were infected for 3 days with colibactin-secreting extra-intestinal pathogenic E. coli (pks+) and its corresponding isogenic mutant (pks-). (A) IL8 secretion was then quantified on cell culture supernatants (Human IL-8/CXCL8 Quantikine ELISA Kit, R&D Systems, Minneapolis, MN, USA) and (B) cells were processed for fluorescent staining with primary antibodies generated against the p65 subunit of nuclear factor κB (NF-κB) and DAPI to counterstain the nuclei (blue). Nuclear translocation of the NF-κB was quantified on a minimum of 500 cells using the "Integrated Density" measure function of ImageJ. *p< 0.005, **p< 0.001, ***p< 0.0001. Abbreviations: AU, arbitrary unit; KO, knock-out; ns, non-significant; pks-, bacterial artificial chromosome vector; pks+, bacterial artificial chromosome vector with pks island encoding colibactin. (PDF) [file ppat.1009320.s007.pdf]

**A)**

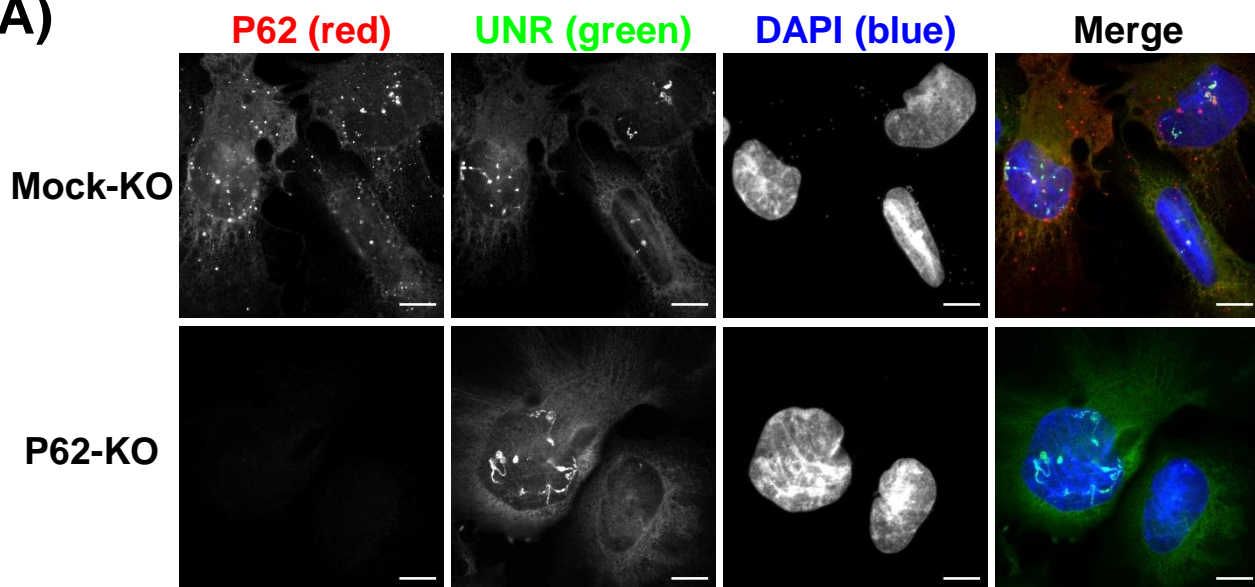

UNR-NR-positive cells (%)

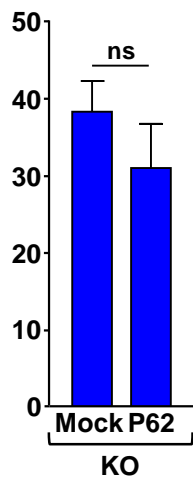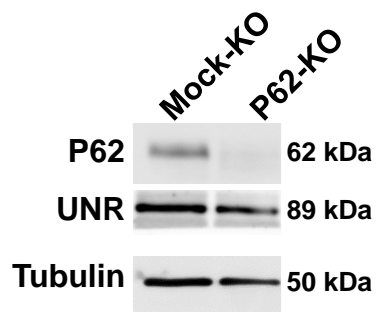

B)

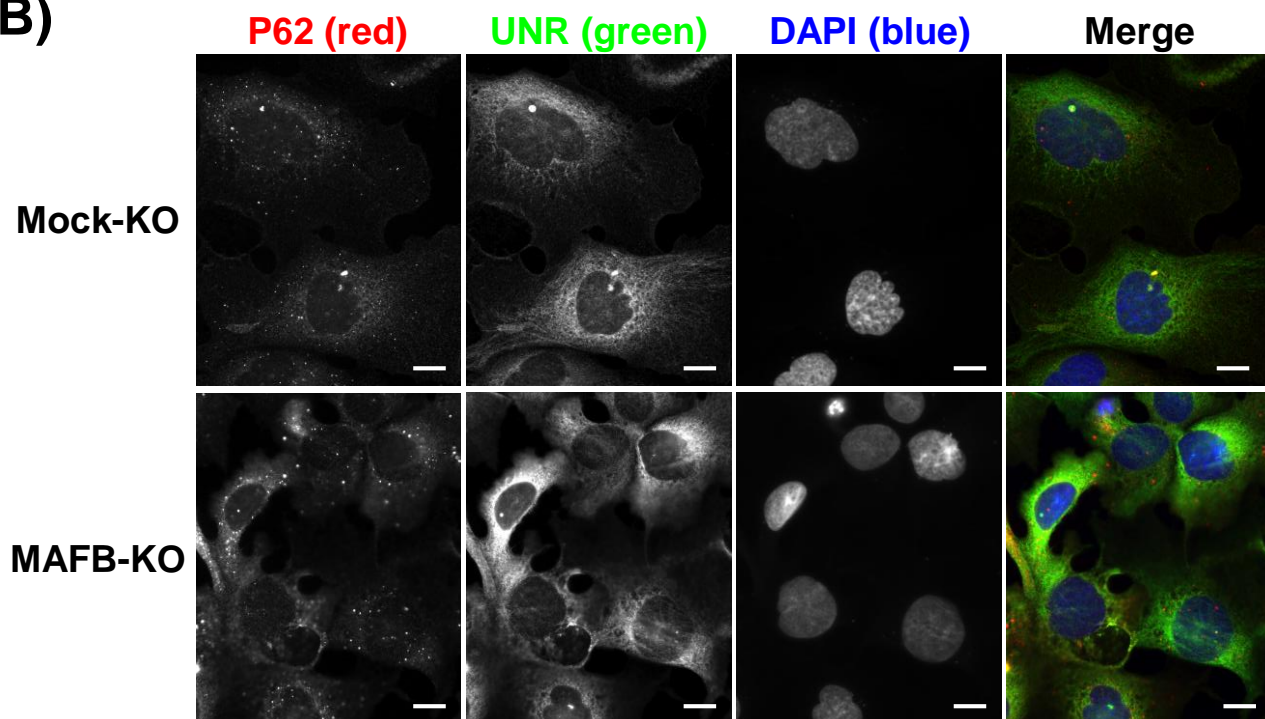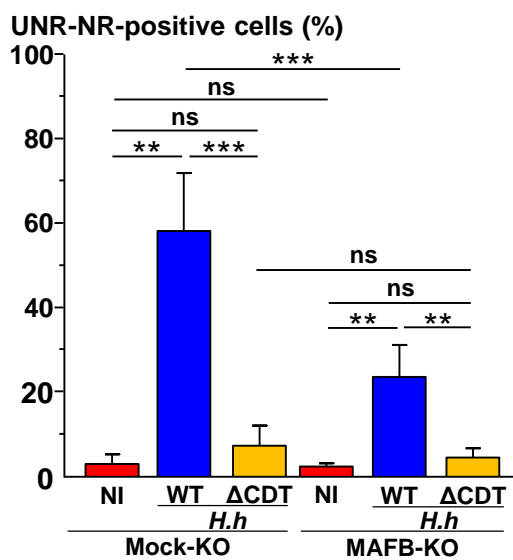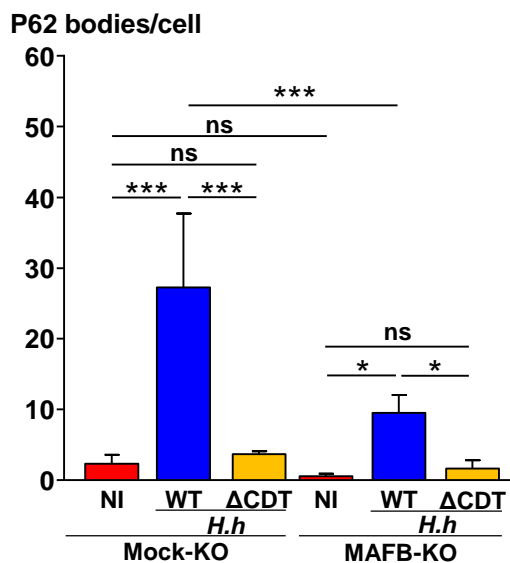

Supplement: S5 Fig — Mock-KO, P62/SQSTM1-KO and MAFB-KO Hep3B cells were not infected or infected for 72 h with H. hepaticus and its corresponding ΔCDT mutant strain. These cells were processed for fluorescent staining with DAPI to detect the nucleus (blue) and primary anti-P62/SQSTM1 (red) and anti-UNR/CSDE1 (green). Fluorescent staining was observed using wide field fluorescence imaging. The percentage of cells presenting UNR-NR was determined by manually counting the number of nuclei displaying UNR spots in the nucleoplasm. P62/SQSTM1 bodies were quantified using the "Find Maxima" function of ImageJ. The results are presented as the mean in one representative experiment (performed in triplicate) out of three. A minimum of 500 nuclei were analyzed. As previously demonstrated, NR formation is primarily observed in response to CDT intoxication, via its active CdtB subunit [6]. Thus, images of non-infected cells are not presented below. (A) Effects of P62/SQSTM1 silencing. Images of cells infected with H. hepaticus: P62/SQSTM1 (red), UNR (green) and DAPI (blue). Scale bar, 20 μm. Quantification of UNR-NR-positive cells in Mock-KO and P62/SQSTM1-KO cells infected with H. hepaticus. Western blot analysis of the protein expression level of P62/SQSTM1, UNR/CSDE1 and tubulin in Mock-KO and P62/SQSTM1-KO Hep3B non infected cells. (B) Effects of MAFB silencing. MAFB silencing was performed as previously reported and lead to a non-clonal cell population comprising 80% of the knock-out (KO) cells for the MAFB gene [33]. Images of in Mock-KO and MAFB-KO cells infected with H. hepaticus: P62/SQSTM1 (red), UNR (green), DAPI (blue). Scale bar, 20 μm. *p<0.05, **p< 0.01, ***p< 0.001. Abbreviations: DAPI, 4′, 6′-diamidino-2-phenylindol; ΔCDT, CDT isogenic mutant of H. hepaticus strain 3B1; H.h, Helicobacter hepaticus; KO, knock-out, NI, non-infected; NR, nucleoplasmic reticulum; ns, non-significant, P62, P62/SQSTM1; WT, H. hepaticus strain 3B1 = wild type strain. (PDF) [file ppat.1009320.s008.pdf]

### Hep3B CdtB

53BP1 (green) + DAPI

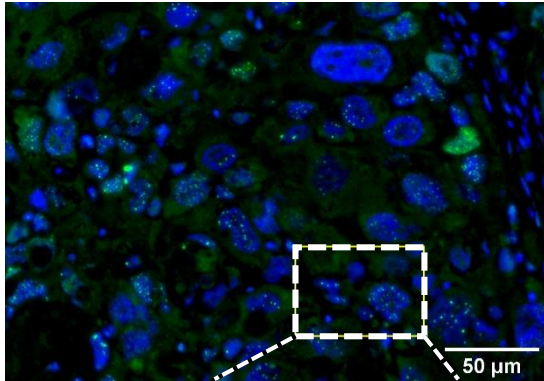

$\gamma$ H2AX (red) + DAPI

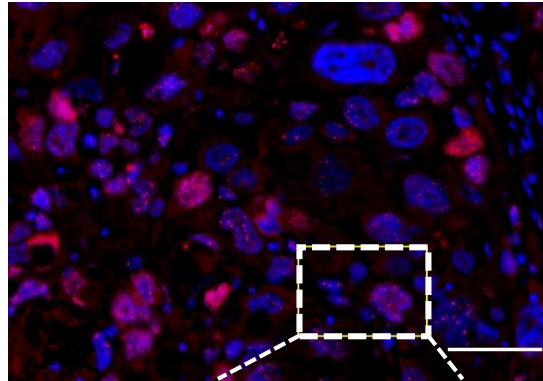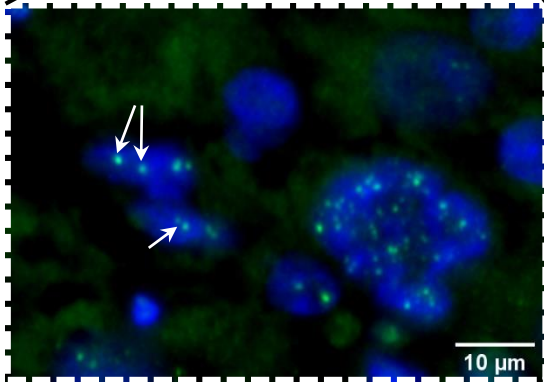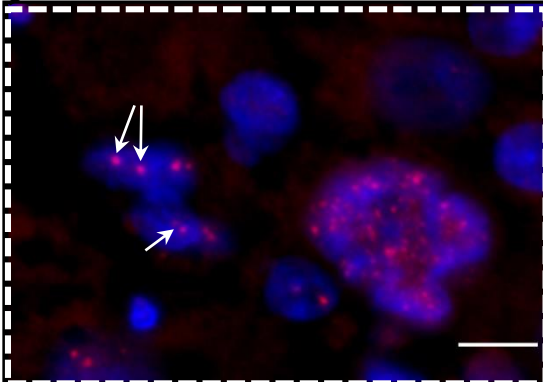

### Hep3B H265L

53BP1 (green) + DAPI

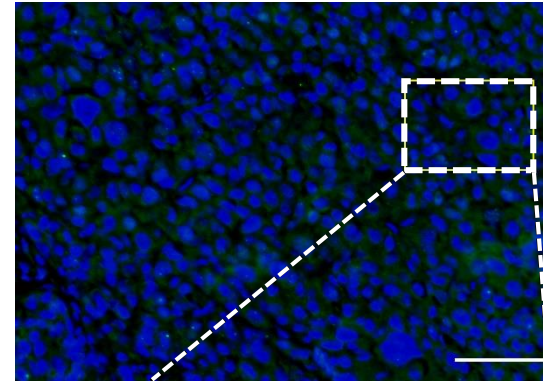

$\gamma$ H2AX (red) + DAPI

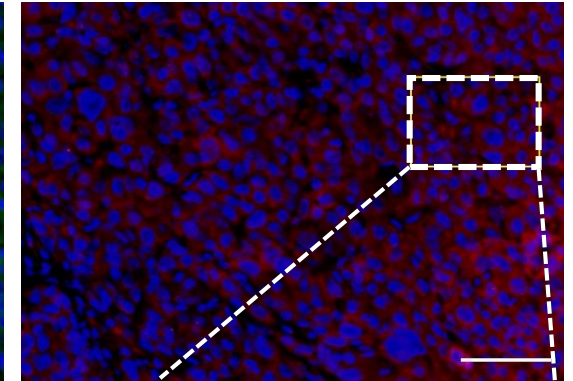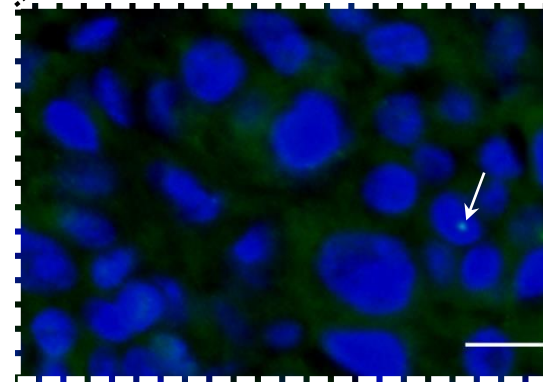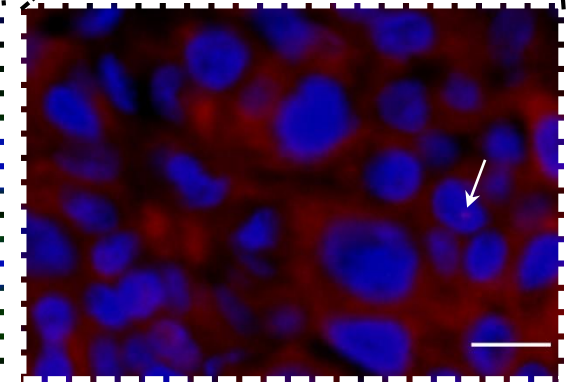

Supplement: S6 Fig — Images of 3-μm-tissue sections of Hep3B-derived mice engrafted tumors sequentially stained for 53BP1 (green) during a first labeling round and γH2AX (red) during a second labeling round, along with a counterstaining with DAPI (blue). Images 53BP-1/DAPI were captured using the microscope (wide field) that records the coordinates of each image, which allows repositioning on the same area after the γH2AX/DAPI immunodetection. Magnifications of selected areas are shown in boxes. Arrows indicates the overlapping of 53BP1 and γH2AX foci. The slight difference between the fields showing the 53BP1 and γH2AX labeling is due to the sequential repositioning between the sequential labeling. Abbreviations: CdtB, CdtB of H. hepaticus strain 3B1; DAPI, 4′, 6′-diamidino-2-phenylindol; H265L, H. hepaticus CdtB with the mutation His→Leu at residue 265 involved in catalytic activity. (PDF) [file ppat.1009320.s009.pdf]
