# Supplementary material for: The CDT of Helicobacter hepaticus induces pro-survival autophagy and nucleoplasmic reticulum formation concentrating the RNA binding proteins UNR/CSDE1 and P62/SQSTM1
Source: PLoS Pathog. 2021 Mar 4;17(3):e1009320. doi: 10.1371/journal.ppat.1009320 (PMC7963068; doi:10.1371/journal.ppat.1009320)
Supplement: S2 Fig — (A) Images of colon HT29 and liver Hep3B following a 72 h doxycycline-induction to induce the expression of the RFP, CdtB of H. hepaticus strain 3B1 and its corresponding mutated CdtB (H265L)[21]. Cells were processed as in Fig 2B1 and 2C1. (B) Images of CdtB- and H265L-expressing colon HT29 and liver Hep3B following a 72 h doxycycline-induction and treatment with bafilomycin A1 or chloroquine. Cells were processed as in Fig 2B2 and 2C2. (C) Images of CdtB- and H265L-expressing colon HT29 and liver Hep3B expressing the tandem-tagged mCherry-GFP-LC3 protein following a 72 h doxycycline-induction. Cells were processed as in Fig 2B3 and 2C3. (D) Autophagic flux was measured following a 72 h doxycycline-induction in CdtB- and H265L-colon SW480 expressing the tandem-tagged mCherry-GFP-LC3 protein with subsequent yellow (mCherry+/GFP+) and red (mCherry+/GFP-) dot/puncta counting (yellow dots) [18]. The results are presented as the mean in one representative experiment (performed in triplicate) out of three. Cells were processed as in Fig 2B3 and 2C3. Images of colon SW480 expressing the tandem-tagged mCherry-GFP-LC3 protein. ***p< 0.001 versus H265L. Scale bar, 20 μm. Abbreviations: DAPI, 4′, 6′-diamidino-2-phenylindol; CdtB, CdtB of H. hepaticus strain 3B1; H265L, H. hepaticus CdtB with the mutation His→Leu at residue 265 involved in catalytic activity; GFP, green fluorescent protein; P62, P62/SQSTM1. (PDF) [file ppat.1009320.s005.pdf]

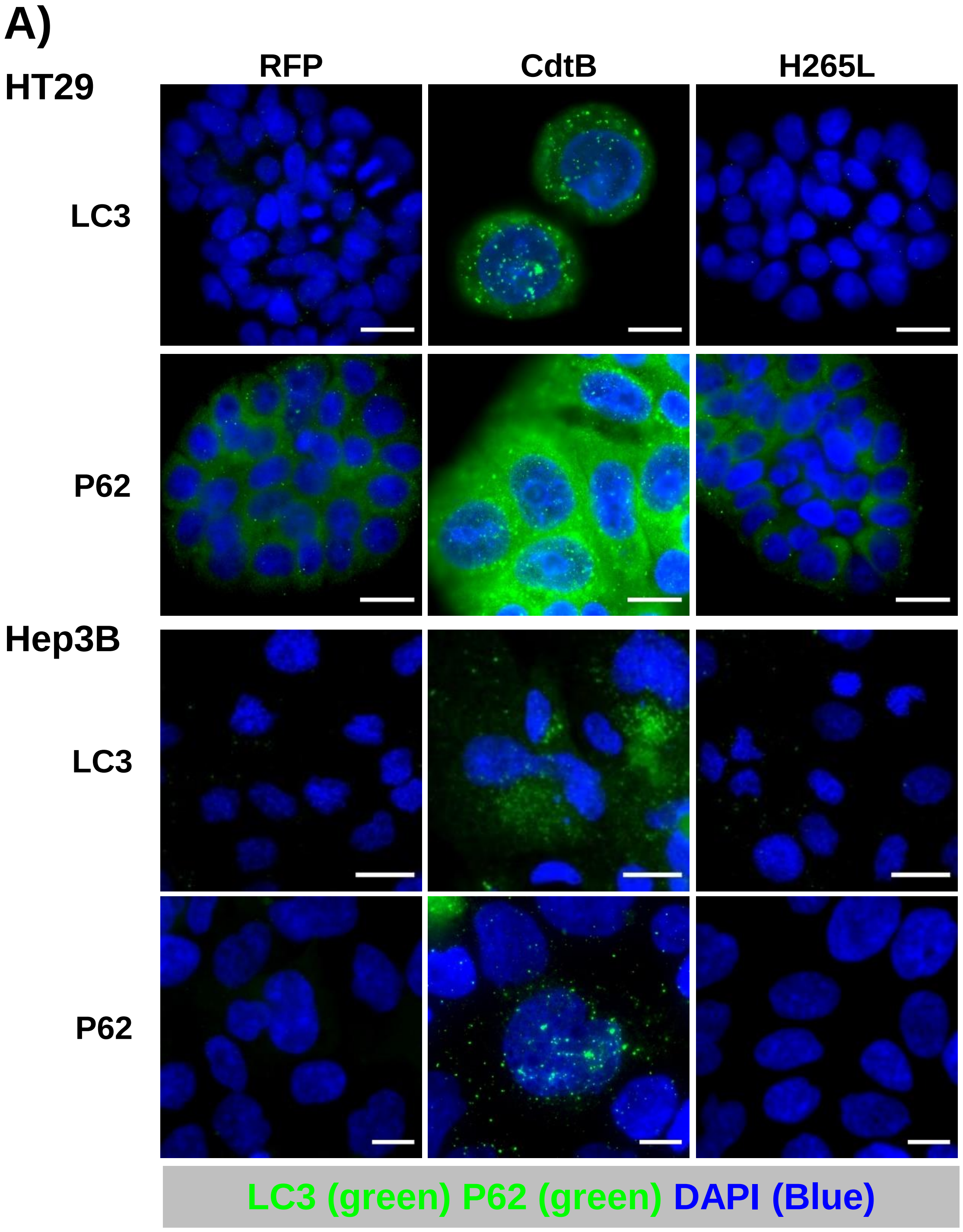
A)
HT29
RFP
CdtB
H265L
LC3
P62
Hep3B
LC3
P62
LC3 (green) P62 (green) DAPI (Blue)

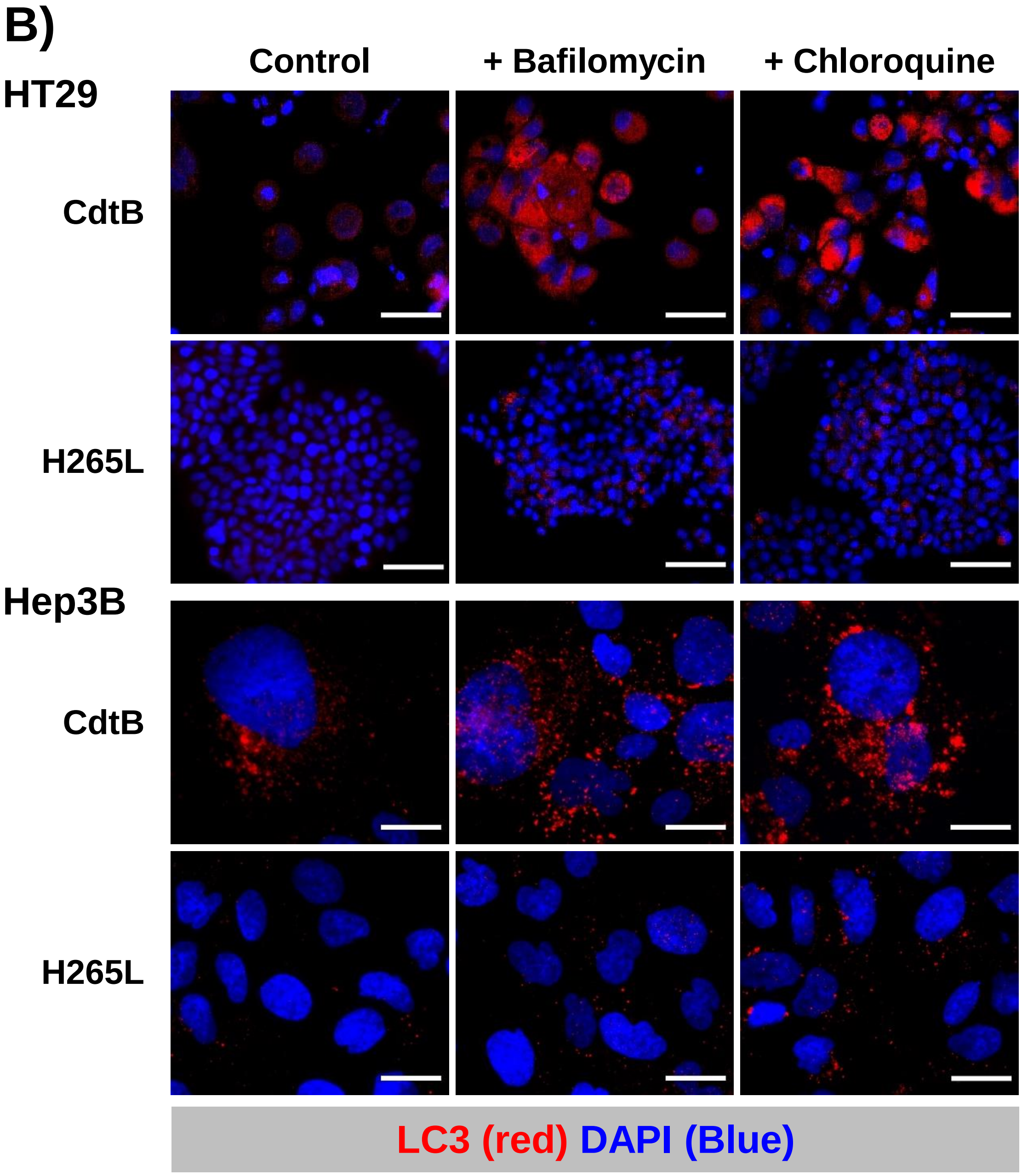
B)
Control
+ Bafilomycin
+ Chloroquine
HT29
CdtB
H265L
Hep3B
CdtB
H265L
LC3 (red) DAPI (Blue)

C)

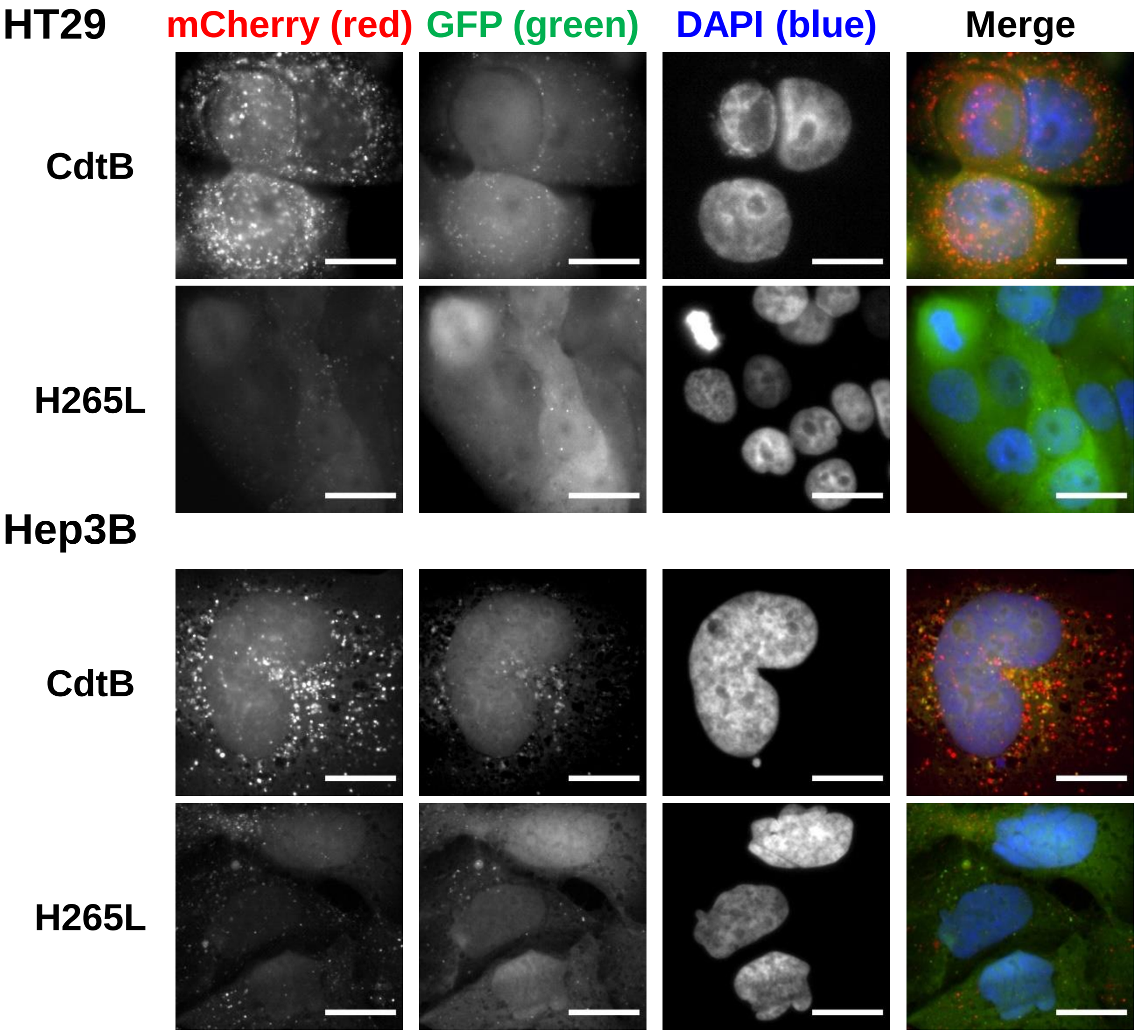
HT29
mCherry (red)
GFP (green)
DAPI (blue)
Merge
CdtB
H265L
Hep3B
CdtB
H265L

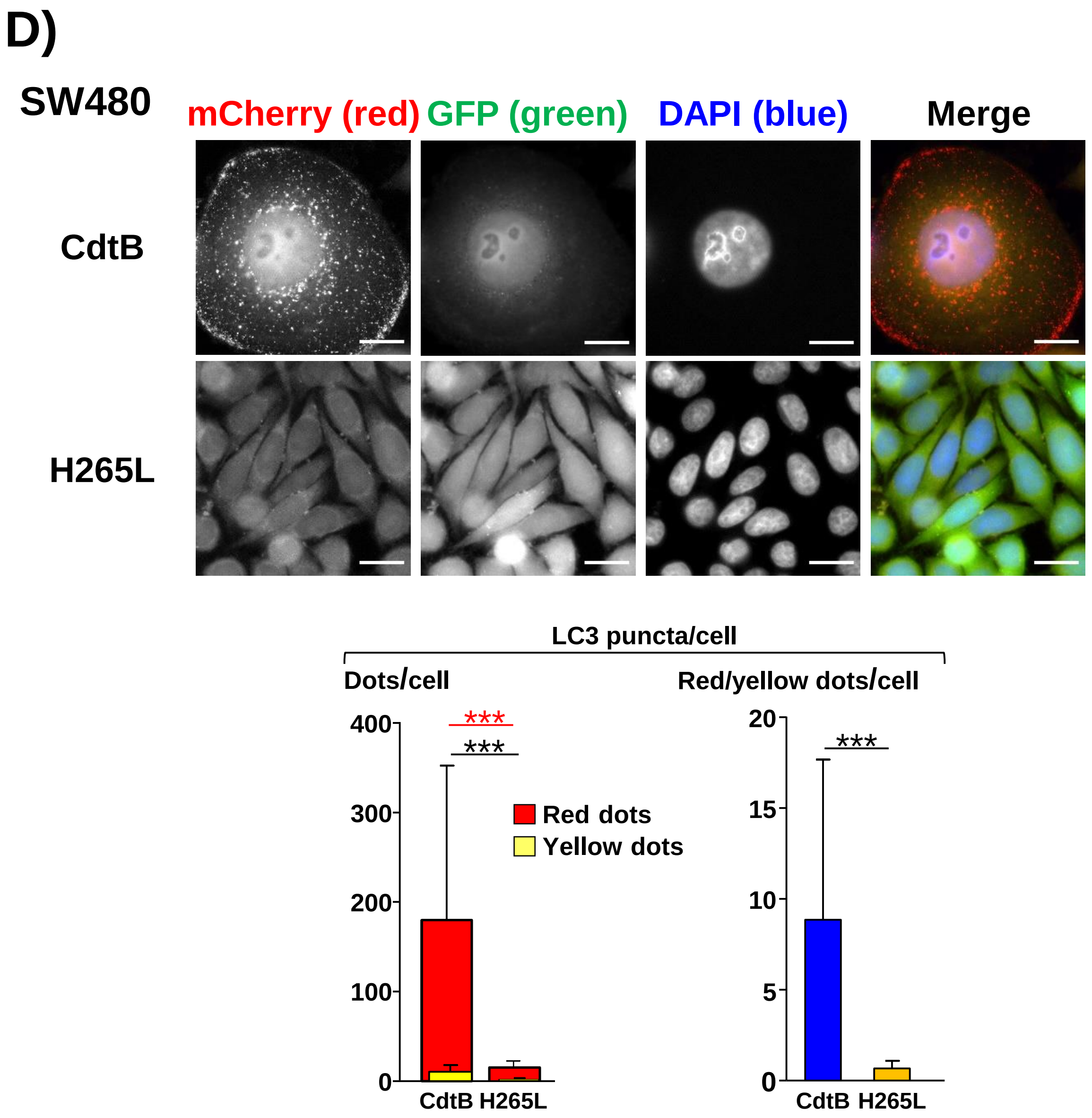

D)
SW480
mCherry (red)
GFP (green)
DAPI (blue)
Merge
CdtB
H265L
LC3 puncta/cell
Dots/cell
Red/yellow dots/cell
400
300
200
100
0
***
***
Red dots
Yellow dots
CdtB H265L
20
15
10
5
0
***
CdtB H265L
