# Supplementary material for: The CDT of Helicobacter hepaticus induces pro-survival autophagy and nucleoplasmic reticulum formation concentrating the RNA binding proteins UNR/CSDE1 and P62/SQSTM1
Source: PLoS Pathog. 2021 Mar 4;17(3):e1009320. doi: 10.1371/journal.ppat.1009320 (PMC7963068; doi:10.1371/journal.ppat.1009320)
Supplement: Attachment — Submitted filename: 2-Reviewers Responses to Questions.pdf [file ppat.1009320.s010.pdf]

Part I - Summary

Reviewer #1: The manuscript entitled ”CDT induces pro-survival autophagy and nucleoplasmic reticulum formation concentrating the RNA binding proteins UNR/CSDE1 and P62/SQSTM1” by He et al describes the activation of autophagy in: i) cells exposed to the H. hepaticus expressing a functional cytolethal distending toxin (CDT) or upon ectopic expression of the active CdtB subunit of the toxin; ii) in in vivo xenograft mouse models. This was assessed by several independent assays ranging from transcriptomic analysis (Figure 1) to immunofluorescence-based methods identifying accumulation of LC3 and p62 punctae in intoxicated/infected cells in vitro or in the xenograft models (Figures 2 and 3).
The authors have identified several nuclear/perinuclear-associated structures which accumulate p62 foci:
i) nucleoplasmic reticulum (NR) invagination, characterized by co-localization of p62 and the UNR protein, devoid of DNA (Figures 3C and 4)
ii) Micronuclei-like structure, positive for LC3 and gH2AX, a surrogate marker of DNA damage (Figures 5A to 5D)
iii) p62 aggregates tightly connected to the lamina of the nuclear membrane (Figure 5E).

Chemical inhibition of autophagy with bafilomycin A/chloroquine and knock out (KO) of ATG5 or ATG7 (key mediators of autophagy) strongly reduces the formation of the UNR-positive structures, reduces gH2AX phosphorylation, increases cell death and formation of p62 punctae (Figure 6).
These effects are not exclusive for CDT intoxication, but they are also induced in cells exposed to Escherichia coli expressing another type of bacterial genotoxin (colibactin, encoded by the pks island, Figure 7), indicating that this is a common response to bacterial genotoxins.

The results reported in this manuscript are interesting and support previous published data demonstrating that CDT induces autophagy and promotes cell survival of the intoxicated cells. In this manuscript the authors associate autophagy activation and cell survival with formation of the p62 positive bodies. The are several issues that need to be clarified. The most relevant is the role of the different p62 positive substructures identified in CDT exposed cells and their role in cell survival, as assessed in more detail in point 1 in Major issues, which will strengthen the biological relevance of the findings.

Reviewer #2: Bacterial genotoxins of the gut microbiota or certain pathogens, including cytolethal distending toxin (CDT), may impact human health. In the current report, whole genome microarrays were performed using HT29 intestinal cells transduced with the active CdtB subunit of Helicobacter hepaticus. The authors observed the CdtB-dependent upregulation of mRNAs involved in positive regulation of autophagy concomitant with the downregulation of mRNAs involved in negative regulation of autophagy. A couple of complimentary studies follow using independent approaches such as immunofluorescence of cell lines and xenograft samples, as well as infection studies with cdtB gene mutants. Together, this is an interesting paper, it’s well written and potentially attractive for a broad readership. I have a few comments that should be considered for improvement.

Reviewer #3: In this work, He et al show in cell culture models of infection with two intestinal pathogens (Helicobacter hepaticus and E coli expressing the pks island and associated genotoxin) that the genotoxic activity is associated with induction of autophagy and the formation of nuclear structures the author refer to as nuclear reticulum, which

contains not only autophagy receptors but also RNA-binding proteins. They show using knock out cell lines lacking key autophagy genes that autophagy is an adaptation protecting the cells against apoptosis upon genotoxic damage.
The experiments are performed with care and the results are clear. This reviewer is a bit mystified by the findings. Is autophagy a general consequence of DNA damage, also with non-microbial stimuli? Could there be additional (non-genome-associated) consequences of CDT or colibactin intoxication that trigger autophagy? The control experiments described below (section 2) will potentially shed light on this problem. Also, the data are not entirely novel, as a previous publication (also in PLOS Pathogens, 2019, same group) has already shown the formation of nuclear reticulum upon CDT and colibactin intoxication.

Part II – Major Issues: Key Experiments Required for Acceptance
Please use this section to detail the key new experiments or modifications of existing experiments that should be absolutely required to validate study conclusions.

Generally, there should be no more than 3 such required experiments or major modifications for a "Major Revision" recommendation. If more than 3 experiments are necessary to validate the study conclusions, then you are encouraged to recommend "Reject".

Reviewer #1: 1. It is not clear from the text whether the structures identified in CDT exposed cells and presented in Figures 3 to 5 are structurally different, and they have in common only the association with p62. Which one is associated with autophagy induction? This is also quite difficult to assess presently, since the authors only report the reduction of UNR positive cells upon inhibition of autophagy (Figure 6). Are these structures directly involved in activation of the survival signals? I understand that proving causality is a very difficult question to assess in this context. However if one of the function of autophagy, induced by CDT, is to remove the micronuclei-like structure and reduce the inflammatory response as proposed by the authors in the Discussion section, then it would important to assess whether there is an increase of the gH2AX/p62 bodies in the ATG5/7 KO cells, and if this correlates with a higher inflammatory response (e.g. higher production of pro-inflammatory cytokine) and with higher levels of activation of caspase 1 (by the inflammasome) leading to pyroptosis (see also point 3). If this can be proven, the activation of autophagy would have two important implications: promote cell survival and reduce the inflammatory response, which would help the CDT-producing bacterium to create a suitable microenvironment to support its survival and replication.
On the same line, it would also be important to evaluate the changes in the UNR/p62 positive and DAPI negative bodies upon inhibition of autophagy, as done for the % of UNR positive cells in Figure 6A.
We agree with these comments and have tried to address them. Antibodies to detect pro-caspase-1 and its cleaved forms are available from limited sources and western blots to detect both isoforms are challenging. We therefore performed immunostaining. The kit for detecting cleaved caspase 1 (Caspase-1 (active) Staining Kit – Green Fluorescence, #ab219935, abcam) failed to provide conclusive results. Cleaved Caspase-1 (Asp297) (D57A2) Rabbit mAb from Cell signaling (#4199) gave some results in 2 publications for Cleaved Caspase-1 immunodetection in cell culture, but not in our experiments.
However, initiator caspases, like caspase-1, do not require cleavage for activation, and so cleavage does not really indicate that the caspase is activated. In addition, enzymatically inactive procaspase 1 stabilizes the pyroptosome (Stein et al. J Biol Chem. 2016 Aug 26;

291(35): 18419–18429). Consequently, we evaluated the rabbit mAb anti-Caspase-1 (D7F10) from Cell signaling (#3866). This antibody gave results only with tissues and showed increased Caspase 1 protein expression in HT29 and Hep3B-derived xenografts in response to CdtB *versus* mutated H265L CdtB (Fig. S1, new panel B). This result is in agreement with microarray data, which showed a 3-fold increase in CASP1 mRNA (as presented in Fig. S1A) concomitant to the activation of autophagy and inflammation in response to CdtB; raw data of inflammation-related genes (TFP and CdtB) presented in Sheet 2 of Table S1.
Interleukin 1β, the substrate of Caspase 1, is weakly expressed in Hep3B and was not detected using the Human IL-1beta ELISA MAX Deluxe kit (Invitrogen, #BLE437004).
On one hand, in an inflammatory microenvironment, caspase-1 is regulated by NF-κB. As CdtB upregulates Caspase 1 mRNA and numerous inflammation-associated genes involved in NF-κB signaling, activates the nuclear translocation of NF-κB and increases IL8 secretion, we quantified NF-κB nuclear translocation and IL8 secretion. See inflammation data in **1)** our previous study (Péré-Védrenne et al, 2016 The Journal of Infectious Diseases 213 (12) 1979-1989) and **2)** raw data of inflammation-related genes (TFP and CdtB) presented in Table S1 (Sheet 2)
IL8 secretion was first quantified using pharmacological inhibitors of autophagy (bafilomycin A1 and chloroquine) in response to CdtB *versus* CdtB H265L. Results obtained with chloroquine were not significant (CdtB *versus* mutated H265L CdtB). In addition, chloroquine induced a slight increase in IL8 secretion in H265L expressing cells, while this compound exhibits a well-known anti-inflammatory effect. On the other hand, Bafilomycin A1 induced an increase in IL8 secretion in H265L expressing cells and this increase was highly significant in response to CdtB. We did not showed these results in the revised manuscript.

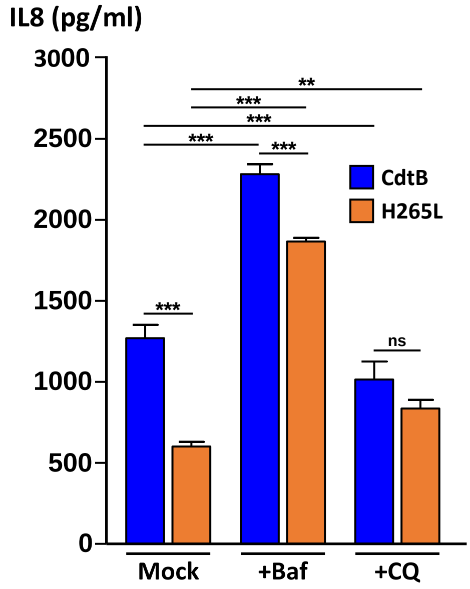

Accordingly, IL8 secretion and nuclear NF-κB were quantified upon infection with *E. coli* secreting colibactin or without colibactin in Mock-, ATG5- and ATG7-KO Hep3B cells. Results using ATG5-KO cell line were not significant. ATG7 silencing resulted in increased IL8 secretion and increased nuclear translocation of NF-κB in response to colibactin, suggesting increased inflammation.
According to this comment, we added Fig. S1B for caspase 1 staining on tissue sections and Fig. S4 for IL8 and nuclear NF-κB quantification. This sentence was added to the 'Discussion' section: 'Preliminary results showed that autophagy disruption by ATG7 silencing led to increased IL8 secretion and nuclear factor κB translocation following bacterial genotoxin

intoxication (Fig. S4A,B), suggesting that autophagy would protect intoxicated cells by reducing inflammatory responses. However, these latest results deserve further study.'

Regarding the quantification of UNR-foci colocalized with p62-bodies in NR, quantification were performed in Mock-, ATG5- and ATG7-KO cells. The number of UNR-foci/p62-bodies in NR increased in reponse to genotoxin upon autophagy disruption: 68.9% in Mock cells, 93.8% in ATG5 cells and 83.9 in ATG5 cells. However, none of these results were significant.

Regarding the quantification of γH2AX-foci/p62-bodies, we had to perform new experiments. As suggested, γH2AX-foci/p62-bodies were quantified in Mock-, ATG5- and ATG7- KO cells and expressed as a percentage of cells positive for both γH2AX-foci/p62-bodies in new panel Fig. 7J. Overall, the number of γH2AX-foci/p62-bodies-positive cells significantly decreased in response to genotoxin when autophagy is disrupted, an expected result as γH2AX-foci strongly decreased upon autophagy disruption in response to bacterial genotoxins.
We also quantified micronuclei-like structures. Disruption of autophagy led to an increase in colibactin-induced micronuclei-like structures, suggesting that they were not removed upon disruption of autophagy (Fig. 7I). These results suggest an autophagic-based selective removal of micronuclei, also called nucleophagy.
A short paragraph was added in "Results" section for these two new panels of Fig. 7.

2. Figures 6G and 6H: p62 levels and p62 punctae in control and ATG5/7 KO cells. The authors states that "P62/SQSTM1, a strong increase in the protein was observed in ATG5- and ATG7-KO infected cells in response to H. hepaticus infection, compared to the Mock-KO cells infected with H. hepaticus" (Results section, page 7, lines 40-41). From the figure shown in Figure 6H, the difference in the levels of p62 expression among the different cells is not clear. In addition, the number of p62 nuclear foci in the ATG 7 cells is similar to the control cells, and much lower than that observed in the ATG5 KO. This does not reflect the data presented in the quantification graph (Figure 6G). The authors should clarify this issue.

This comment is pertinent. The text written in the manuscript is correct. There was an increase in P62 at the basal level in ATG5 and ATG7 and this increase was more important in response to CDT. Indeed, the pictures do not represent the results. According to this comment, new photographs were taken and all of the photographs were changed. Moreover, the ATG5-CDT and ATG7-CDT curves were inverted during the manual annotation of squares and triangles. It is now correct, as other minor errors. Thank you so much.

3. Discussion section. The author state that "H. hepaticus CdtB also regulated some genes involved in the 'apoptosis and autophagy' pathway and the increase in the mRNA level of apoptosis-regulator proteins and inflammatory caspases suggests CdtB-induced pyroptosis during H. hepaticus infection" (Page 11, lines 23-25). Conversely, the data in presented in this manuscript support a role of autophagy in survival of the CDT exposed cells, therefore I was wondering how these two opposite outcomes can be reconciled.

This comment is pertinent. Some studies showed that, despite the marked differences between apoptosis and autophagy, their regulation is intimately connected and both of these two supposedly different processes can be stimulated by the same stresses, as is the case in the present study with CDT and colibactin (Figs 6 & 7). Under certain circumstances, autophagy constitutes a stress adaptation that protects cells from various apoptotic stimuli, whereas in other

cellular settings, it constitutes an alternative cell-death pathway (reviewed in Thorburn Apoptosis. 2008; 13(1): 1–9).
Both autophagy and apoptosis were induced following CDT and colibactin intoxication. We can assume that autophagy constitutes an adaptation to the genotoxic stress induced by CDT and colibactin, which counteracts the genotoxin-induced apoptosis/pyroptosis to maintain cell survival during DNA damage repair. However, for cells whose DNA damage is beyond repair, autophagy might not be sufficient to maintain cell survival leading to apoptotic cell death or autophagy might induce apoptotic cell death. A paragraph was added in the "Discussion section".

Reviewer #2:

1.) The M&M section should contain a separate paragraph describing in detail the microarrays, which is missing. I also think the overall microarray data should be available to the readers, and not only a subset (shown in Fig. 1). Please deposit these data in the public domain, for example at the GEO database. They give you an accession number that can be provided to the reviewers and later to the readers. You can embargo this information for the public domain until the paper is published.

As requested, we have added a paragraph "Transcriptomics" in the "Materials and Methods" section. Raw data for genes involved in autophagy, including those that are not expressed or regulated by CdtB, has been added in a new supplementary Table S1 (sheet 1). We do not wish to give access to all of the MicroArray data as these data are the basis of other studies in progress. However, the raw data for genes known to be regulated during inflammation (714 genes, 1146 annotations) are presented in Table S1 (sheet 2).

2.) The biological significance of the findings in the paper are not clear to me. It seems not really obvious what is the link between the nuclear bodies positively stained for UNR-p62 or p62/gH2AX related to autophagy-associated cell survival caused by CdtB? Eventually, this could reduce the overall inflammatory reaction (seen by the microarrays or cytokine ELISA?) and could block cell death, which helps the infection strategy of the intruding pathogen? This question could be tackled by some experiments, which in turn can provide biological importance to the studies.

We agree with these comments and have tried to address them. We tried to evaluate inflammation upon autophagy inhibition. However, results with Chloroquine and ATG 7-KO cells were not conclusive.

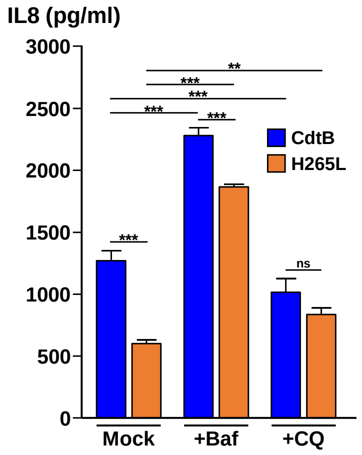

Nevertheless, we showed that disruption of autophagy by ATG7 silencing led to increased IL8 secretion and NF-κB translocation following colibactin intoxication (new Fig. S4A,B), suggesting that autophagy would protect intoxicated cells by reducing inflammatory responses. According to this comment, we added Fig. S4 for IL8 and NF-κB translocation quantification and a sentence was added to the "Results section":

Regarding the quantification of γH2AX-foci/p62-bodies, we had to perform new experiments. As suggested, γH2AX-foci/p62-bodies were quantified in Mock-, ATG5- and ATG7- KO cells and expressed as a percentage of cells positive for both γH2AX-foci/p62-bodies in new panel Fig. 7J. Overall, the number of γH2AX-foci/p62-bodies-positive cells significantly decreased in response to genotoxin when autophagy is disrupted, an expected result as γH2AX-foci strongly decreased upon autophagy disruption in response to bacterial genotoxins.

We also quantified micronuclei-like structures. Disruption of autophagy led to an increase in colibactin-induced micronuclei-like structures, suggesting that they were not removed upon disruption of autophagy (see new panel in Fig. 7I). These results suggest an autophagic-based selective removal of micronuclei, also called nucleophagy. A short paragraph was added in "Results" section for these two new panels of Fig. 7.

Taken together, these results suggest that autophagy would protect intoxicated cells by reducing inflammatory responses and removal of damaged DNA.

Reviewer #3: Major points:

1. Please use one or two positive controls of DNA damage and genomic instability as controls throughout. Topoisomerase inhibitors or etoposide, or hydroxyurea, or UV irradiation might all work. It is important to see whether any kind of DNA damage leading to DNA DSBs will trigger autophagy and nuclear reticulum formation, or whether this is a specific adaptation to bacterial genotoxins. The best control would be another alkylating agent, which would mimic the effects of colibactin.

According to this comment, we evaluated the effect of DNA damaging molecules with different mechanisms of action: etoposide, a topoisomerase II enzyme inhibitor, and streptozotocin, an antimicrobial agent used as a chemotherapeutic alkylating agent. NR formation concentrating UNR and P62 and autophagy were also observed in Hep3B cells showing that nucleoplasmic reticulum formation and autophagy are not restricted to CDT and colibactin, but also occur in response to other non-alkylating and alkylating DNA damaging agents.

Moreover, these data suggest that nucleoplasmic reticulum formation and autophagy may contribute to the resistance of cancer cells to therapies inducing DNA damage, opening new fields of investigations. Indeed, altered nuclear morphology reminiscent of NR is a common feature of many cancers and has adverse prognostic significance for various cancers (see Malhas et al. Trends Cell Biol. 2011; 21(6):362–373). Two paragraphs and a new Fig. 8 were added in the "Results" and "Discussion" sections, as well as a sentence in the abstract. Thank you for this comment.

2. the authors use micronuclei formation as a readout of genomic instability. The most important question is however not addressed with this assay. How does the inhibition of autophagy (in the ATG5 and ATG7 ko) affect micronuclei formation? In this same context, did the authors expect to see reduced yH2AX foci in cells incapable of autophagy? If DNA DSBs are upstream of autophagy induction, does this finding make sense? Not really to this

reviewer. A proper quantification of micronuclei (as a fraction of all binucleated cells) is required.

We understand this comment. In the present study, micronuclei formation was not used as a readout of genomic instability. It is indeed pertinent to quantify micronuclei-like structures and γH2AX-foci/p62-bodies in cells incapable of autophagy.

Regarding the quantification of γH2AX-foci/p62-bodies, we had to perform new experiments. As suggested, γH2AX-foci/p62-bodies were quantified in Mock-, ATG5- and ATG7- KO cells and expressed as a percentage of cells positive for both γH2AX-foci/p62-bodies in new panel Fig. 7J. Overall, the number of γH2AX-foci/p62-bodies-positive cells significantly decreased in response to genotoxin when autophagy is disrupted, an expected result as γH2AX-foci strongly decreased upon autophagy disruption in response to bacterial genotoxins.

We also quantified micronuclei-like structures. Disruption of autophagy led to an increase in colibactin-induced micronuclei-like structures, suggesting that they were not removed upon disruption of autophagy (Fig. 7I). These results suggest an autophagic-based selective removal of micronuclei, also called nucleophagy.

A short paragraph was added in “Results” section for these two new panels of Fig. 7. Thank you for this comment.

3. gH2AX as the only readout of DNA DSBs is questionable. Please include a second marker (53BP1 or other) to ascertain that the gH2AX signal is indeed indicative of DNA damage. It doesn't look "focal" enough to this reviewer.

We understand this comment. γH2AX is the most commonly used DSB biomarker, but can be observed in response to other stresses. Accordingly, we performed successive new staining with rabbit anti-53BP1 and rabbit anti-γH2AX on the same slide. As both antibodies originated from rabbits, sequential fluorescent labeling was performed using 3-µm tissue sections, prepared from formalin-fixed paraffin-embedded human CdtB-Hep-3B xenografts. Firstly, 53BP1 immunodetection was performed, images (53BP1/DAPI) were captured and coordinates of each image were recorded to ensure repositioning of the slide to the same area after γH2AX labeling. Secondly, γH2AX immunodetection was performed using the same slide; the images (γH2AX/DAPI) were captured using the repositioning function of the microscope (Zeiss Axioplan 2 fluorescence microscope, Zeiss, Jena, Germany).

Overall, cells presenting γH2AX foci upon CdtB expression also have 53BP1 foci and conversely, while cells without γH2AX foci do not have 53BP1 foci, showing that γH2AX signal is indeed indicative of DNA damage, strengthening the fact that CDT induces DSB. These results were previously reported in other publications (Blazkova et al. 2010 J Cell Mol Med. 14: 357–367; Fedor et al. 2013 Cell Microbiol. 15:1-15; Fahrer et al. DNA Repair 2014, 18: 31-43).

A Supplementary Fig. S5 showing the overlapping of 53BP1 and γH2AX foci in response to CdtB was added, as well as the description of the successive labeling in the section “Material & Methods”.

Part III – Minor Issues: Editorial and Data Presentation Modifications

Please use this section for editorial suggestions as well as relatively minor modifications of existing data that would enhance clarity.

Reviewer #1:

1. In Figure3C, it is not really possible to judge whether these structures are external to the nuclear compartment and devoid of DNA, since the DAPI staining is not shown.
As requested, DAPI staining was added. Accordingly, RGB staining and the corresponding merge are in revised Fig. 3C.

2. Figure 5, it would be good to have a higher magnification of the gH2AX/p62 bodies. A higher magnification of all bodies presented in Fig. 5 was added on the right panel of Fig. 5. Moreover, yellow and pink boxes with increased staining were added (when the staining was weak).

3. Figure 5 E is not commented in the text, and also for this figure a higher magnification of the structures indicated would help the reader.
As mentioned previously, a higher magnification was added on the right panel of the Fig. 5E. Fig. 5E was referred at the end of the corresponding sentence in the revised manuscript: "P62/SQSTM1 large bodies/aggregates were also found tightly connected to the lamina of the nuclear membrane, and the nuclear lamina seemed to engulf these aggregates (Fig. 5E)."

Reviewer #2:

1.) The name of the bacterium "Helicobacter hepaticus" should be in the title of the paper.
Helicobacter hepaticus was added in the title.

2.) Figures 2 and S4: Please add size markers to all Western blots.
The molecular weight of each protein was added to all Western blots.

Reviewer #3: please make sure all axes are properly labeled for easy comprehension of figure content without the need to read the entire legend.
All axes (especially in Fig. 6) are properly labeled.
