# Supplementary material for: The CDT of Helicobacter hepaticus induces pro-survival autophagy and nucleoplasmic reticulum formation concentrating the RNA binding proteins UNR/CSDE1 and P62/SQSTM1
Source: PLoS Pathog. 2021 Mar 4;17(3):e1009320. doi: 10.1371/journal.ppat.1009320 (PMC7963068; doi:10.1371/journal.ppat.1009320)
Supplement: Attachment — Submitted filename: Round 2 - Reviewers Responses to Questions.pdf [file ppat.1009320.s011.pdf]

Dear Dr. Menard,

Thank you very much for submitting your manuscript "The CDT of Helicobacter hepaticus induces pro-survival autophagy and nucleoplasmic reticulum formation concentrating the RNA binding proteins UNR/CSDE1 and P62/SQSTM1" for consideration at PLOS Pathogens. As with all papers reviewed by the journal, your manuscript was reviewed by members of the editorial board and by several independent reviewers. The reviewers appreciated the attention to an important topic. Based on the reviews, we are likely to accept this manuscript for publication, providing that you modify the manuscript according to the review recommendations.

Please prepare and submit your revised manuscript within 30 days. If you anticipate any delay, please let us know the expected resubmission date by replying to this email.

When you are ready to resubmit, please upload the following:

[1] A letter containing a detailed list of your responses to all review comments, and a description of the changes you have made in the manuscript.

Please note while forming your response, if your article is accepted, you may have the opportunity to make the peer review history publicly available. The record will include editor decision letters (with reviews) and your responses to reviewer comments. If eligible, we will contact you to opt in or out

[2] Two versions of the revised manuscript: one with either highlights or tracked changes denoting where the text has been changed; the other a clean version (uploaded as the manuscript file).

Important additional instructions are given below your reviewer comments.

Thank you again for your submission to our journal. We hope that our editorial process has been constructive so far, and we welcome your feedback at any time. Please don't hesitate to contact us if you have any questions or comments.

Sincerely,

Steffen Backert
Guest Editor
PLOS Pathogens

Guy Tran Van Nhieu
Section Editor
PLOS Pathogens

Kasturi Haldar
Editor-in-Chief
PLOS Pathogens
orcid.org/0000-0001-5065-158X

Michael Malim
Editor-in-Chief
PLOS Pathogens
orcid.org/0000-0002-7699-2064

Reviewer Comments (if any, and for reference):

Reviewer's Responses to Questions

Part I - Summary

Please use this section to discuss strengths/weaknesses of study, novelty/significance, general execution and scholarship.

Reviewer #1: The authors have satisfactorily replied to most of the questions risen during the first revision. Importantly they have shown preliminary data demonstrating that inhibition of autophagy can be associated with increased production of inflammatory mediators, supporting an interesting line of investigation concerning the link between bacterial genotoxins and reduction of the host inflammatory response.

Reviewer #2: I appreciate very much the revised paper. The authors have satisfactorily commented to my points.

Part II – Major Issues: Key Experiments Required for Acceptance

Please use this section to detail the key new experiments or modifications of existing experiments that should be absolutely required to validate study conclusions.

Generally, there should be no more than 3 such required experiments or major modifications for a "Major Revision" recommendation. If more than 3 experiments are necessary to validate the study conclusions, then you are encouraged to recommend "Reject".

Reviewer #1: (No Response)

Reviewer #2: (No Response)

Part III – Minor Issues: Editorial and Data Presentation Modifications

Please use this section for editorial suggestions as well as relatively minor modifications of existing data that would enhance clarity.

Reviewer #1: There is still one issue which is not clear to me: it is very difficult to assess from the representative micrograph presented in Figure 6H whether there is a decreased of gH2AX in infected cells upon ATG5 or ATG7 knock out (KO). If this is the case, then this would suggest that inhibition of autophagy is associated with alteration of the DNA damage response. It would be good if the authors:

1) show a lower magnification of the representative gH2AX staining micrographs so the reader can appreciate the reduction in the amount of H2AX phosphorylation in the KO cells in Figure 6H.

According to this comment, we added a lower magnification in Fig. 6H. (X10 instead of X40). Scale was changed 100 µm for γH2AX and DAPI double staining (see legend of Fog. 6H).

2) add in the discussion a sentence concerning the possible mechanisms that promote decreased H2AX phosphorylation upon inhibition of autophagy.

This sentence was added:
"In the present study, γH2AX foci and nucleus size were measured in viable and early apoptotic cells; thus, their global decrease in response to CDT and colibactin upon autophagy disruption did not reflect their overall cellular quantification but likely reflects the enhanced apoptotic cell death. Moreover, it is also possible that autophagy disruption conducted to activation of alternative DDR pathways (instead of the canonical pathway) [27], which functioned as backup and processed the genotoxin-induced DNA breaks, resulting in γH2AX foci and nucleus size decrease. Further investigation is needed to explore the DDR molecular regulation in autophagy-deficient cells in the context of bacterial genotoxin response."
According to this comment, a new reference was added, ref [27]; and the legend of Figs 6 and 7 was modified.

Reviewer #2: (No Response)

PLOS authors have the option to publish the peer review history of their article (what does this mean?). If published, this will include your full peer review and any attached files.

If you choose "no", your identity will remain anonymous but your review may still be made public.

Do you want your identity to be public for this peer review? For information about this choice, including consent withdrawal, please see our Privacy Policy.

Reviewer #1: No

Reviewer #2: No

Figure Files:

While revising your submission, please upload your figure files to the Preflight Analysis and Conversion Engine (PACE) digital diagnostic tool, https://pacev2.apexcovantage.com. PACE helps ensure that figures meet PLOS requirements. To use PACE, you must first register as a user. Then, login and navigate to the UPLOAD tab, where you will find detailed instructions on how to use the tool. If you encounter any issues or have any questions when using PACE, please email us at figures@plos.org.

Data Requirements:

Please note that, as a condition of publication, PLOS' data policy requires that you make available all data used to draw the conclusions outlined in your manuscript. Data must be deposited in an appropriate repository, included within the body of the manuscript, or uploaded as supporting information. This includes all numerical values that were used to generate graphs, histograms etc.. For an example see here: http://www.plosbiology.org/article/info%3Adoi%2F10.1371%2Fjournal.pbio.1001908#s5.

Reproducibility:

To enhance the reproducibility of your results, PLOS recommends that you deposit laboratory protocols in protocols.io, where a protocol can be assigned its own identifier (DOI) such that it can be cited independently in the future. For instructions see http://journals.plos.org/plospathogens/s/submission-guidelines#loc-materials-and-methods
